# α-synuclein fibrils *per se* but not α-synuclein seeded aggregation causes mitochondrial dysfunction and cell death in human neurons

**DOI:** 10.1016/j.redox.2025.103817

**Published:** 2025-08-10

**Authors:** Plamena R. Angelova, Noemi Esteras, James Evans, Marko Kostic, Ronald Melki, Jochen H.M. Prehn, Sonia Gandhi, Andrey Y. Abramov

**Affiliations:** a Department of Clinical and Movement Neurosciences, UCL Queen Square Institute of Neurology, London, UK; b Neurochemistry Research Institute, Department of Biochemistry and Molecular Biology, School of Medicine, Complutense University of Madrid, Spain; c CIBERNED, Network Center for Biomedical Research in Neurodegenerative Diseases, Spain; d TEVA Pharmaceuticals, Israel; e Institut Francois Jacob (MIRCen), CEA, CNRS, Fontenay-aux-Roses, France; f Department of Physiology and Medical Physics, SFI Future Neuro Research Centre, RCSI University of Medicine and Health Sciences, Dublin, Ireland

**Keywords:** *α-synuclein*, *Seeding*, *Snca*, *Parkinson's disease*, *Neurotoxicity*

## Abstract

One of the major histopathological features of Parkinsons's disease – intracellular Lewy bodies - consists of misfolded α-synuclein. This protein can self-assemble, spread through the brain and seed its own aggregation. Aggregated α-synuclein is shown to induce mitochondrial dysfunction that leads to neuronal loss. Using human iPSC-derived SNCA triplication (3xSNCA) and isogenic control (ISO) neurons we studied whether acute exposure to fibrillar α-synuclein, or its seeding properties, induce effects on mitochondrial function and toxicity. Chronic exposure of neurons to fibrillar α-synuclein (up to 3 weeks) induces a gradual increase of endogenous α-synuclein seeding in neurons, with a decrease in the exogenous fibrillar α-synuclein in ISO and 3xSNCA neurons. Application of exogenous fibrillar α-synuclein induced mitochondrial depolarisation, impairment of complex I function, increased ROS production, oxidative stress and cell death. Notably, α-synuclein seeding following weeks of incubation almost completely restored mitochondrial function and redox balance of human neurons. Thus, mitochondrial dysfunction and oxidative stress in human neurons can be induced acutely only by transient exogenous fibrillar α-synuclein, but seeding is irrelevant to long-term mitochondrial dysfunction or toxicity. This study also indicates an acute, transient toxic insult followed by a remarkable period of adaptation and functional recovery, highlighting the resilience of human neurons.

## 1 Introduction

Described more than 200 years ago for the first time, Parkinson's disease remains a devastating and incurable disorder nowadays. The pathology of Parkinson's disease is associated with loss of dopaminergic neurons in *substantia nigra* and intracellular inclusions termed Lewy bodies rich in aggregated α-synuclein (aSyn) [1]. Importantly, mutations in the SNCA gene leads to familial forms of Parkinson's disease, thus α-synuclein, both as a gene and a protein, is closely associated with sporadic and familial forms of Parkinson's disease [2].

Physiological form of aSyn is thought to be the monomeric form that plays a role in the regulation of synaptic transmission, dopamine signalling and mitochondrial energy metabolism [[3], [4], [5]]. However, misfolding of aSyn and its ability to form aggregates that spread and multiply [1] by seeding the aggregation of monomeric α-synuclein is believed to be a trigger for pathology. Aggregation of aSyn into oligomers and fibrils is mainly connected to the pathology and turning mechanisms on that lead to neurodegeneration. Different aggregate forms of α-synuclein are involved in abnormal calcium signalling in neurons and astrocytes [[6], [7], [8]], ROS overproduction and oxidative stress [[9], [10], [11]]. Mitochondria are heavily involved in the mechanisms underlying Parkinson's disease pathology and different aggregate species of α-synuclein have specific effects on mitochondrial function and shape. Thus, fibrillar and oligomeric α-synuclein are able to inhibit complex I [[12], [13], [14]], and/or change mitochondrial membrane potential and ATP production [4,15]. Application of oligomeric aSyn also activates autophagy and mitophagy in control and cells with familial forms of Parkinson's disease and changes their mitochondrial dynamics [16,17].

Pathogenic aggregated aSyn assembled *de novo* or from patient brains is shown to have the ability to spread between cells, and cellular transmission leads to seeded aggregation in the recipient neurons [18]. A major question in the field remains whether α-Syn-induced toxicity, or whether aSyn-induced seeding and aggregation, is the critical mechanism driving the pathogenesis of Parkinson's disease. Changes in mitochondrial function is one of the most profound phenotypes induced by aggregated aSyn. Thus, we used human cortical iPSC-derived isogenic controls and neurons with *snca* multiplication (iPSC-3.2 A) differentiated from NPCs [19] to assess the effect of fibrillar p91 aSyn on mitochondrial function, seeding and neuronal cell death. We report that exogenous p91 accumulates in neurons in the first 3 days of application followed by a slow clearance of these aggregates over the following 3 weeks of observation. Seeding of p91 became noticeable after 72 h and the number of neurons with p91 seeding increased with time over the following 3 weeks. Importantly, acute application of p91 induces changes in mitochondrial membrane potential, reduction of ATP, mitochondrial ROS production and decrease in GSH. However, prolonged incubation of the iPSC-derived neurons with p91 fibrils induced seeding of α-synuclein but recovery of mitochondrial function. Thus, seeding of fibrillar aSyn in control human neurons and neurons with SNCA triplication does not result in mitochondrial dysfunction or neuronal cell death.

## 2 Materials and methods

### 2.1 p91 fibrils preparation

Human wild-type aSyn, expressed in *E. coli* BL21 DE3 CodonPlus cells and purified as described in Ref. [20], was dialyzed overnight at 4 °C against 1000 vol of 20 mM NaP04, 150 mM KCl, pH 9.1. Monomeric a-Syn (300 μM) was incubated at 37 °C under continuous shaking in an Eppendorf Thermomixer set at 600 r.p.m. for 7 days. Assembly into p91 fibrils was monitored by thioflavin T binding using a Cary Eclipse Fluorescence Spectrophotometer (Varian Medical Systems Inc.) using an excitation wavelength = 440 nm, an emission wavelength = 480 nm. The resulting fibrils were imaged by Transmission Electron Microscopy (TEM) after adsorption of the fibrils onto carbon-coated 200 mesh grids and negative staining with 1 % uranyl acetate using a Jeol 1400 transmission electron microscope and the images were recorded via a Gatan Orius CCD camera (Gatan, Pleasanton). Their characteristic proteolytic pattern was checked using digestion with proteinase K. P91 fibrils were centrifuged twice at 50,000g for 15 min and re-suspended twice in PBS. Their concentration was adjusted to 350 μM in PBS. They were next fragmented by sonication for 20 min in 2 mL Eppendorf tubes using a Vial Tweeter powered by an ultrasonic processor UIS250v (250 W, 2.4 kHz; Hielscher Ultrasonic) to an average length of 50 nm. The fragmented fibrils were aliquoted (6 μL) in Eppendorf tubes, flash frozen in liquid nitrogen and stored at −80 °C until use. Fibrils labelling with ATTO-488 NHS-ester was achieved by adding 2 M equivalents of the extrinsic fluorophore in DMSO to unfragmented fibrils. After incubation, at room temperature for 1h, the fibrils were centrifuged, re-suspended in PBS and fragmented as described above.

Experiments on penetration, distribution of P91- ATTO-488 were controlled in separate experiments with ATTO-488 only to avoid misinterpretation.

### 2.2 Neuronal differentiation

NPCs were generated by the Broccoli Lab as previously described with appropriate optimization [21]. For differentiation, NPCs were dissociated with Accutase and plated on Matrigel-coated six-well plates (1 × 300,000 cells per well) in NPC medium. Two days after, the differentiation medium containing Neurobasal (Thermo Fisher Scientific), Pen/Strep (1 %), B27 (1:50), with SU5402 (Sigma-Aldrich, 10 μM), PD0325901 (Sigma-Aldrich, 8 μM), DAPT (Sigma-Aldrich, 10 μM) was added and kept for 2 days. The differentiation medium was replaced every day with a fresh one on days 1 and 2. On day 3, cells were detached by Accutase solution incubation at 37 °C for 10 min and centrifuged, counted, and seeded onto poly-l-lysine/laminin/fibronectin (all from Sigma-Aldrich, 100 μg/ml, 2 μg/ml, 2 μg/ml)-coated plates in neuronal maturation medium supplemented with ROCK inhibitor Y27632 (10 μM). Neuronal maturation medium was composed of Neurobasal A (Thermo Fisher Scientific) supplemented with 1 × B27 supplement, 2 mM glutamine, 1 % Pen/Strept, BDNF (Peprotech, 20 ng/ml), ascorbic acid (Sigma-Aldrich, 100 nM), Laminin (1 μg/μl), DAPT (10 μM), and dbcAMP (Selleckchem, 250 μM). The culture medium was replaced after 24 h to remove the ROCK inhibitor, and then half of the medium was replaced with a fresh neuronal maturation medium twice a week. After 10 days, cells were dissociated with Accutase and plated on poly-l-lysine/laminin-coated 24-well plates for the final maturation. BDNF (10 ng/ml), GDNF (10 ng/ml), DAPT (10 μM, Sigma-Aldrich), and Ascorbic Acid (10 μM, Sigma-Aldrich) were added from day 20 onwards to promote neuronal maturation and survival [19]. The density of the cells was not increase in the time of all 3 weeks of experiments that prove the absence of proliferation.

### 2.3 Immunocytochemistry

Cells were fixed for 20 min in iced 4 % paraformaldehyde (PFA, Sigma), diluted in phosphate-buffered saline (PBS, Gibco). Then, cells were permeabilized for 30 min in a blocking solution, containing 0.5 % Triton X-100 (Sigma-Aldrich) and 10 % donkey serum (Sigma-Aldrich), incubated overnight at 4 °C with the primary antibodies in a blocking solution. Then, cells were washed with PBS and incubated for 1 h at room temperature with Hoechst and with secondary antibodies. The following antibodies were used: anti-α-Synuclein (clone LB509, 1:100, Thermo Fischer), anti-α-Synuclein (phosphoS129, 1:300, Abcam), and a donkey secondary antibody used for the immunofluorescence staining was Alexa Fluor 555-labelled.

### 2.4 Mitochondrial membrane potential and mitochondrial fragmentation

To visualize mitochondrial membrane potential and shape, neurons were incubated for 40 min with 25 nM tetramethylrhodamine methyl ester (TMRM) in HBSS buffer. TMRM was maintained in the solution for the duration of the acquisitions. Z-stacks were acquired using a Zeiss 710 VIS CLMS confocal microscope equipped with a META detection system and an x40 oil immersion objective (Zeiss, Oberkochen, Germany). TMRM was excited with the 561 nm laser line and emitted fluorescence was measured above 580 nm. For mitochondrial membrane potential measurements, TMRM intensity was analysed in individual cells (neuronal bodies, n = 50–153) using Fiji software. Mitochondrial Network Analysis (MiNA) toolset was used to analyse the mitochondrial network shape and fragmentation using Fiji software following the creators’ protocol [22]. The median length of all mitochondrial rods and branches and the number of branches per network were analysed in a total of 5–16 images in different independent experiments. Neuronal bodies were manually excluded from the analysis, and only axonal mitochondrial were considered.

### 2.5 Seahorse experiments

IPSC-derived neurons plated in Neurobasal media at a seeding density of 3 x 10^4^ cells per well. Seahorse XF Assays were conducted according to the manufacturer's instructions (Agilent Technologies), where the Seahorse XF Cell Mito Stress test kit (Agilent Technologies) was used to perform the experiments using the Seahorse XFe24 Analyzer. All reagents used in this assay were obtained from Agilent Technologies. Oligomycin (1 μM), FCCP (1 μM) and rotenone/antimycin A (1 μM) were used for the experiments and the XF Cell Mito Stress Test template protocol in the Agilent Wave Software program was selected to perform the experiments.

### 2.6 NADH autofluorescence measurements

NADH autofluorescence was measured using an epifluorescence inverted microscope equipped with a × 40 fluorite objective. Excitation light at a wavelength of 360 nm was provided by a Xenon arc lamp, the beam passing through a monochromator (Cairn Research, Faversham, Kent, UK). Emitted fluorescence light was reflected through a 455 nm long-pass filter to a cooled CCD camera (Retiga, QImaging) and digitized to 12-bit resolution. Imaging data were collected and analysed using Andor iQ3 software (Belfast, UK).

### 2.7 Lipid peroxidation assay

The rate of lipid peroxidation was measured using confocal microscopy. Confocal images were obtained with a Zeiss LSM 710 with an integrated META detection system. To assess lipid peroxidation C11-BODIPY (581/591, 2 μM, Molecular probes) was excited using the 488 and 543 nm laser line and fluorescence measured using a band-pass filter from 505 to 550 nm and 560 nm long-pass filter (40 × objective). Illumination intensity was kept to a minimum (at 0.1–0.2 % of laser output) to avoid phototoxicity, and the pinhole set to give an optical slice of ∼2 μm. Addition of a bright field image allowed separation of neurons, that are visibly distinguishable and are situated on different focal plane. Data were acquired and analysed using ZEN2009 software.

### 2.8 H_2_O_2_ levels measurements

For the intracellular H_2_O_2_ assessments using HyPer-3 indicator, human iPSC-derived neurons were transfected with the Hyper-3 construct or the Hyper-CS control pH probe using Effectene according to the manufacturers' instructions (QIAGEN). Importantly, due to the toxicity of the Effectene reagent in combination with Neurobasal A media, cells were exposed to the transfection complexes for an hour, and the media was replaced with fresh Neurobasal A medium. Cells were then left to express the constructs for 48 h before experiments were performed. Hyper-CS or HyPer-3 were excited at 488 and 405 nm and emission were set to 510–540 nm and values expressed as 488/405 ratio [11].

### 2.9 ATP level measurements

To determine the ATP levels, IPSC-derived neurons were transfected with a mitochondrially-targeted genetically-encoded ATP probe (AT1.03), using Effectene according to the manufacturer's instructions (Qiagen). The FRET signal was quantified by the 527:475 nm ratio following an excitation of 405 nm and a filter from 515 to 580 nm [23].

### 2.10 Mitochondrial ROS production measurement

For measurement of mitochondrial ROS production, cells were preincubated with MitoTracker Red CM-H(2)XROS for 10 min at RT. MitoTracker Red CM-H(2)XROS measurements were produced using 560 nm excitation and emission above 580 nm. The indicator irreversibly oxidised and become fluorescent and the measurement of the rate of oxidation was used for identification of production of ROS in the matrix of mitochondria.

### 2.11 Glutathione levels

To measure the levels of GSH a confocal microscope (ZEISS 710 with an integrated META detection system) was used. GSH levels were measured using Monochlorobimane (MCB; 50 μM) loaded for 30 min in RT.

#### 2.11.1 Cell death assay

Quantification of cell death was performed using high-throughput imaging in 96-well plate format. Prior to imaging cells were washed with Hank's balanced salt solution before being loaded with 10 μM Hoechst and 500 nM SYTOX green. Live-cell images were acquired using an Opera Phenix High-Content Screening System (PerkinElmer). SYTOX green and Hoechst were imaged using the 488 and 405 nm laser respectively. 20 fields of view were taken for each well with 5 z-stacks. Extraction of data from the Columbus Studio Cell Analysis Software was performed using an established pipeline [24].

### 2.12 Caspase-3 activation

For measurements of caspase-3 activation cells were loaded for 15 min at room temperature with 10 μM NucView 488 caspase-3 substrate (Biotium, USA) in HBSS. The substrate can rapidly cross cell membrane to enter the cell cytoplasm, where it is cleaved by caspase-3 to release the high-affinity DNA dye. The released DNA dye migrates to the cell nucleus to stain the nucleus brightly green. To estimate the total number of cells with activated caspase-3, calcium ionophore 100 μM Ferutinin was added to induce mitochondrial permeability transition pore opening and trigger activation of apoptosis [25,26].

Confocal images were obtained using Zeiss (Oberkochen, Germany) 710 confocal laser scanning microscope and a 40 × oil immersion objective. The 488 nm argon laser was used to excite NucView 488 fluorescence, which was measured using a bandpass filter from 510 to 560 nm.

### 2.13 Statistics

All values are expressed as mean ± SEM. Differences between means were analysed using the Student's t-test, one-way or two-way analysis of variance (ANOVA) depending on the number of groups and variables in each experiment. Data was then submitted to Tukey or Bonferroni post hoc test using Origin Pro software. The null hypothesis was rejected when the P value was <0.05. The choice of statistical test has been stated in each figure legend.

### 2.14 Data availability

Raw data that support the findings in this study are available from the corresponding author upon reasonable request.

## 3 Results

### 3.1 p91 α-synuclein fibrils penetrate human iPSC-derived neurons and induce seeding

**Fig. 1:**
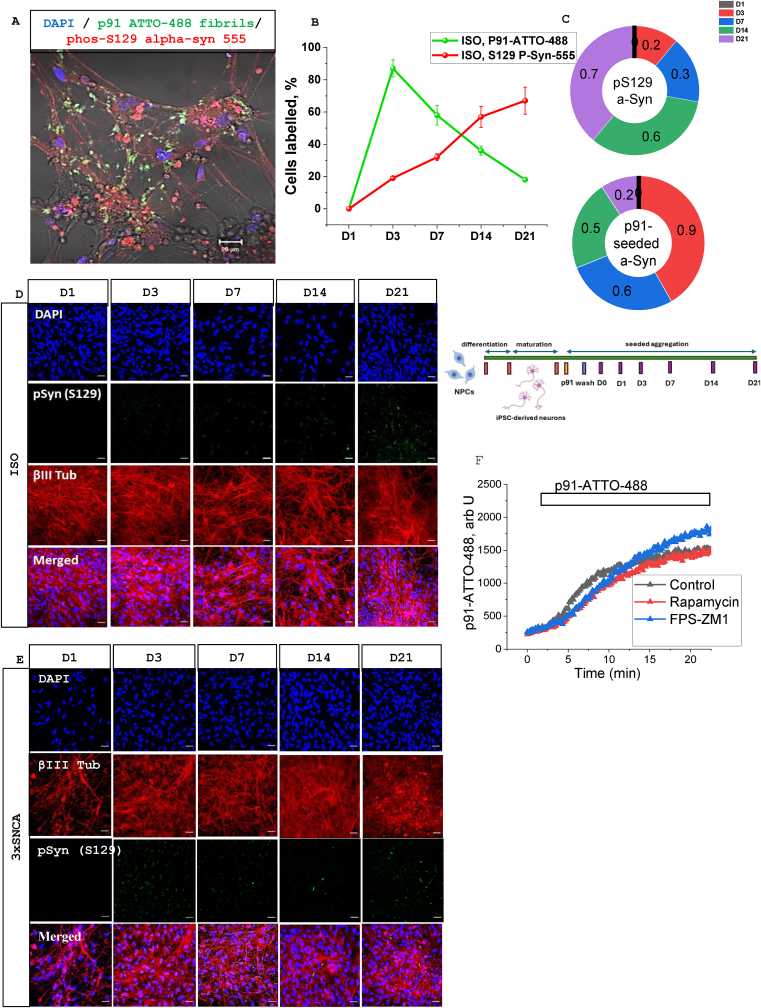
**Time-dependent α-synuclein spreading and seeding in control and 3xSNCA in human cortical iPSC-derived neurons. A** – Confocal images showing localization of P91-ATTO-488 fibrils, immunostaining for hyper-phosphorylated aSyn (pSyn(S129)) and DAPI in control neurons 3 days post incubation with P91 fibrils. Percentage of control human neurons with P91-ATTO-488 fibrils (green curve) and phosphorylated aSyn (pSyn(S129), red curve) inclusions as a function of time after application are presented in (**B**) and as fractions of seeded (upper panel) vs. phosphorylated aSyn (lower panel) over time (clockwise) in ISO neurons (**C**). The number of cells labelled with phosphorylated aSyn (pSyn(S129)) Ab increased with time after application of P91 fibrils in control (ISO, **D**) and SNCA triplication (3xSNCA, **E**) neurons. Inset: scheme depicting protocols for differentiation if IPSCs, seeding with p91 and timing of imaging. Values are mean ± SEM of N = 3–5 independent differentiation batches (n = 50–250 ROI for each group). Scale bars, 20 μm. F- The changes in levels of p91-ATTO-488 fibrils fluorescence in ISO neurons after application of fibrils (see bar) in untreated (control) cells and neurons treated with 100 nM rapamycin or 1 μM FPS-ZM1.

In order to assess if p91 aSyn fibrils are able to enter cells, we applied fluorescently labelled p91 fibrils to control human iPSC-derived neurons. This resulted in an increase of fluorescence and distribution of the p91 fibrils inside the cells 30 min after application (Fig. 1 A). Importantly, fluorescence of the labelled p91 fibrils was observed in the cells after 1, 3-, 7-, 14- and 21-days post exposure, although the levels of exogenous p91 fibrils gradually decreased with time (Fig. 1 B; N = 3 experiments for each measurement).

One of the important characteristics of misfolded proteins is not only their ability to spread through cells (Fig. 1 A) but also their induction of seeding [27]. Incubation of human neurons with labelled or non-labelled p91 fibrils induces seeding (detected with phospho-S129 aSyn-555 antibodies) in both human control and neurons (β-tubulin antibody positive) with SNCA triplication already on the 3rd day after aSyn application (Fig. 1B and C-D). However, the number of cells with aSyn seeding increased over time and was maximal after 21 days of observation (Fig. 1B). Interestingly, signals from exogenous α-synuclein p91 and phospho-aSyn (seeded) did not always co-localise inside of IPSC-derived human neurons (Fig. 1A).

Several ways to transport extracellular α-synuclein were suggested including the mTOR pathway and interaction with the RAGE receptor [28,29]. Using pre-treatment of the neurons with rapamycin (1 h, 100 nM; N = 3) or a RAGE receptor inhibitor, 1 μM FPS-ZM1 (20 min; N = 3), which successfully blocked RAGE in our previous study [30]– we estimated the role of these pathways on the fluorescent p91 fibrils uptake (Fig. 1 F). It should be noted that both – rapamycin and FPS-ZM1 did not change the uptake kinetics of fluorescent p91 into the cells compared to untreated neurons suggesting that fibrillar p91 is transported into the cells by another mechanism.

Thus, fibrillar p91 aSyn is able to penetrate the neurons and induce seeding.

### 3.2 Acute but not chronic application of p91 α-synuclein fibrils induces mitochondrial depolarisation

**Fig. 2:**
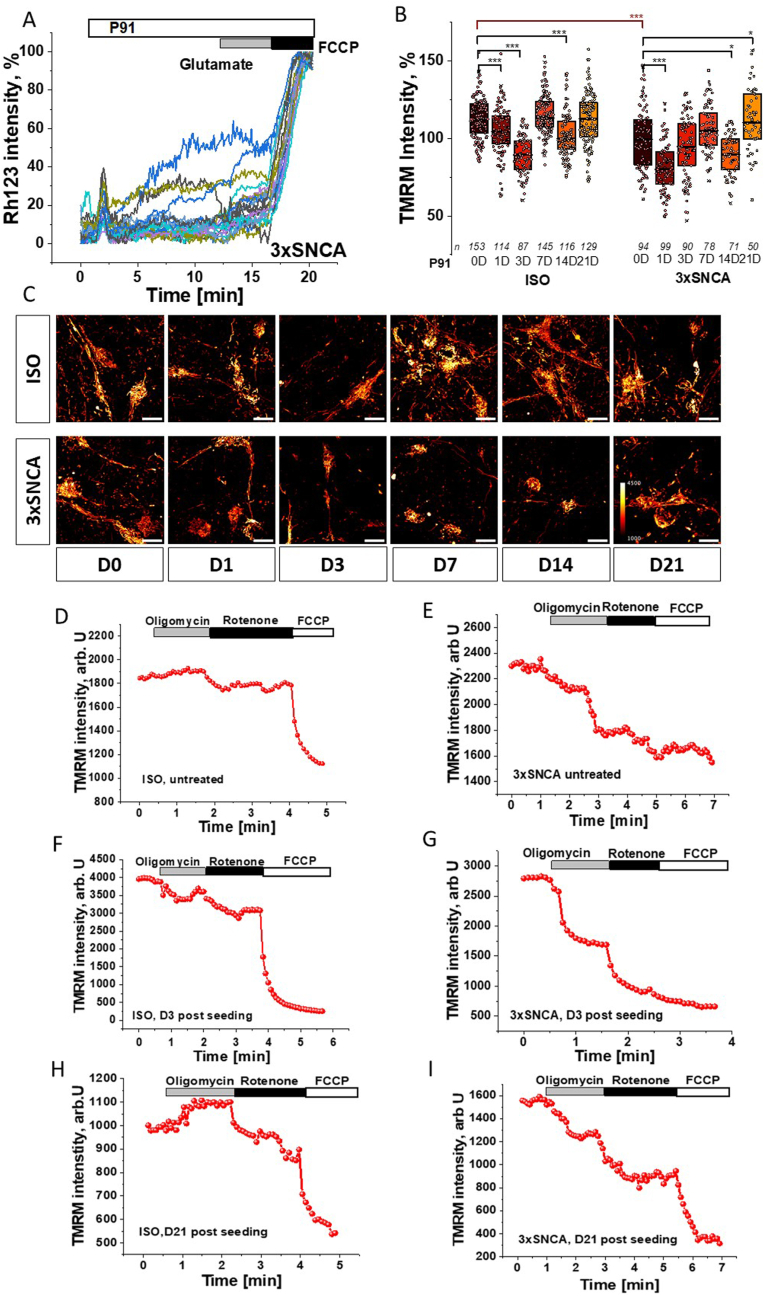
**Effect of p91 fibrils spreading and seeding on mitochondrial membrane potential in control and 3xSNCA human iPSC-derived neurons.** A- Application of p91 aSyn induced changes in Rh123 fluorescence in neurons with SNCA triplication. Glutamate 10 μM, 1 μM FCCP. B- mitochondrial membrane potential (TMRM fluorescence, normalized to untreated ISO control) in days after p91 application. n= number of neurons analysed showed as dots in 3–4 experiments. Box plots represent the median and 25 and 75 percentiles. Non-parametric Kruskal-Wallis ANOVA with post-hoc Dunn's test for each group, Mann-Whitney for CTR vs. SNCA at D0, ∗p < 0.05, ∗∗p < 0.01, ∗∗∗p < 0.0001.C – representative images of TMRM fluorescence in control and 3xSNCA neurons over time after P91 application. Scale bar: 10 μm. Color-coded scale bar shown in last image. D-I Changes in TMRM fluorescence in response to oligomycin (2 μg/ml), rotenone (5 μM) and 1 μM FCCP in untreated control and 3xSNCA neurons and in 3 and 21 days after p91 application. Values I the representative traces are mean ± SEM of N = 3–5 independent differentiation batches (n = 50–153 ROI for each group).

Similarly to reported for other fibrils and oligomeric aSyn [14,15], application of p91 α-synuclein fibrils to iPSC-derived control neurons loaded with a probe for mitochondrial membrane potential (Δψm) – Rh123, induced slow mitochondrial depolarisation in the majority of neurons (*67 ± 7 %, n=255 cells*) with profound (up to *60 %* of Rh123 signal, Fig. 2A) mitochondrial depolarisation in a small proportion of the neurons (*8–12 %* of cells). As previously described for oligomeric aSyn [15] the mitochondrial membrane potential of SNCA triplication neurons was significantly lower than in controls basal conditions (Fig. 2B–C). Incubation of the neurons with the p91 aSyn fibrils led to further significant decrease in Δψm at shorter incubation times followed by a recovery of Δψm over the following 3 weeks (Fig. 2B–C). Dysfunction of the mitochondrial electron transport chain causing mitochondrial depolarisation can be compensated by other mechanisms [31]. In control neurons application of inhibitor of F0–F1-ATPsynthase (2 μg/ml oligomycin) induced either an increase or no effect on TMRM fluorescence, suggesting that this enzyme is working as ATP synthase (Fig. 2 D). Subsequent application of inhibitor of mitochondrial complex I (5 μM rotenone) reduced Δψm confirming the role of this complex in the maintenance of Δψm. Application of uncoupler (1 μM FCCP) leads to a complete mitochondrial depolarisation, indicating that a relatively large proportion of Δψm in these cells is maintained by the activity of ETC starting from complex II (Fig. 2D). In agreement with previously published data [15] in neurons with SNCA triplication, Δψm is maintained partially by F0–F1-ATPase (reverse mode) activity as oligomycin-induced a decrease in TMRM fluorescence in these cells (Fig. 2 E). It should be noted that 72 h incubation of the ISO control and SNCA triplication neurons with p91 aSyn fibrils changed the direction of the response to oligomycin in control cells and further increased oligomycin-induced mitochondrial depolarisation in cells with SNCA triplication (Fig. 2F–G), suggesting induction of mitochondrial dysfunction in neurons at this stage. However, the response to oligomycin in both ISO control and SNCA triplication neurons was restored on the day 21 compared to the pathology induced in initial days of p91 application (Fig. 2H–I). Thus, application of p91 fibrils-induced changes in mitochondrial membrane potential and the mechanism of maintenance of Δψm in the first days of application, but after more prolonged incubation recovery is observed.

### 3.3 α-synuclein seeding changes mitochondrial NADH redox balance

**Fig. 3:**
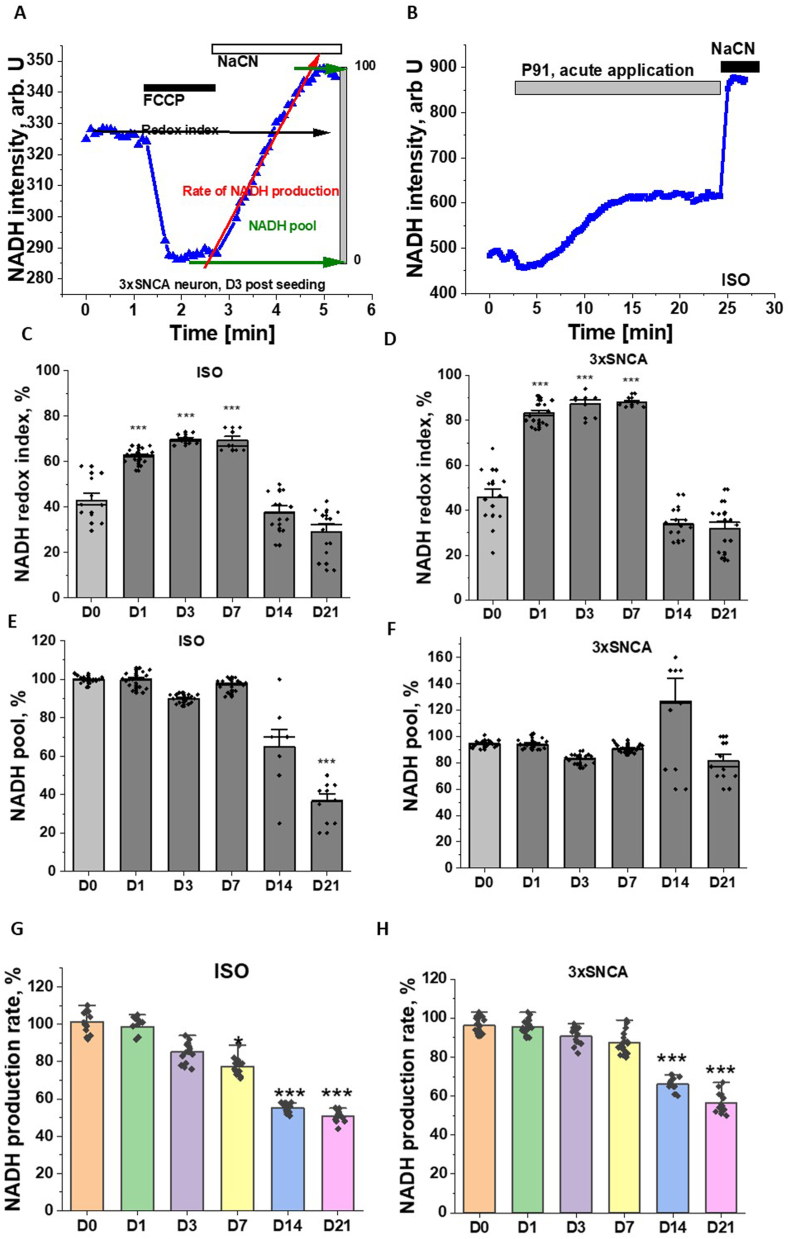
**p91 α-synuclein fibrils spreading and seeding change mitochondrial NADH redox balance in control and 3xSNCA human iPSC-derived neurons.** A- Changes in NADH autofluorescence in response to 1 μM FCCP and 1 mM NaCN in 3xSNCA neurons 3 days after p91 application given as example for calculation method of redox index, NADH level and NADH production rate. B- acute application of p91 to control neurons induced increase in NADH autofluorescence. Changes in mitochondrial redox index in ISO control (C) and 3xSNCA neurons (D) after p91 application as a function of time. Changes in mitochondrial NADH level in ISO control (E) and 3xSNCA neurons (F) after p91 application as a function of time. Changes in the rate of NADH production in ISO control (G) and 3xSNCA neurons (H) after p91 application as a function of time. Values are mean ± SEM of N = 3 independent differentiation batches (n = 150 ROI for each group). Statistical analysis is performed using one-way ANOVA followed by Tukey post-test. ∗p < 0.05, ∗∗p < 0.01, ∗∗∗p < 0.0001.

NADH is a major donor of electrons to mitochondrial complex I and measurements of NADH autofluorescence provides information about mitochondrial respiration and enzyme activity in live cells. Mitochondrial NADH fluorescence can be distinguished from NADPH signal and non-mitochondrial NADH using 1 μM FCCP which maximises respiration in cells (minimising mitochondrial NADH level, which is taken as 0 %) followed by inhibition of respiration by 1 mM NaCN, which blocks consumption of NADH and maximally increases NADH level in mitochondria (Fig. 3 A). The difference in NADH between minimal and maximal values represents the mitochondrial NADH pool, the initial level of NADH in relation to maximal and minimum is a balance between NADH production and consumption and can be described as NADH redox index. Additionally, the rate of NADH autofluorescence increase after inhibition of respiration can indicate the rate of NADH production in TCA cycle (Fig. 3A). In agreement with our previous results [14], application of fibrillar aSyn p91 induced a slow and progressive increase in mitochondrial NADH level, suggesting partial inhibition of mitochondrial complex I (Fig. 3B). Prolonged incubation of the ISO control neurons and SNCA triplication to p91 induced an increase of NADH redox index in the first 7 days of exposure, suggesting inhibition of complex I-dependent respiration (Fig. 3C–D). However, the p91-induced increased NADH redox index was not observed on day 14 and 21. It was further not associated with an increase in the NADH pool (Fig. 3E–F). Actually, incubation of SNCA triplication cells did not significantly change mitochondrial NADH pool for all days of measurements (Fig. 3 F) but in ISO control neurons incubation with p91 induced a decrease in mitochondrial NADH pool beyond day 7 that can be possibly explained by the inhibition of NADH production in mitochondria on these days (Fig. 3G and H).

### 3.4 SNCA triplication but not α-synuclein seeding inhibits mitochondrial respiration and ATP production

**Fig. 4:**
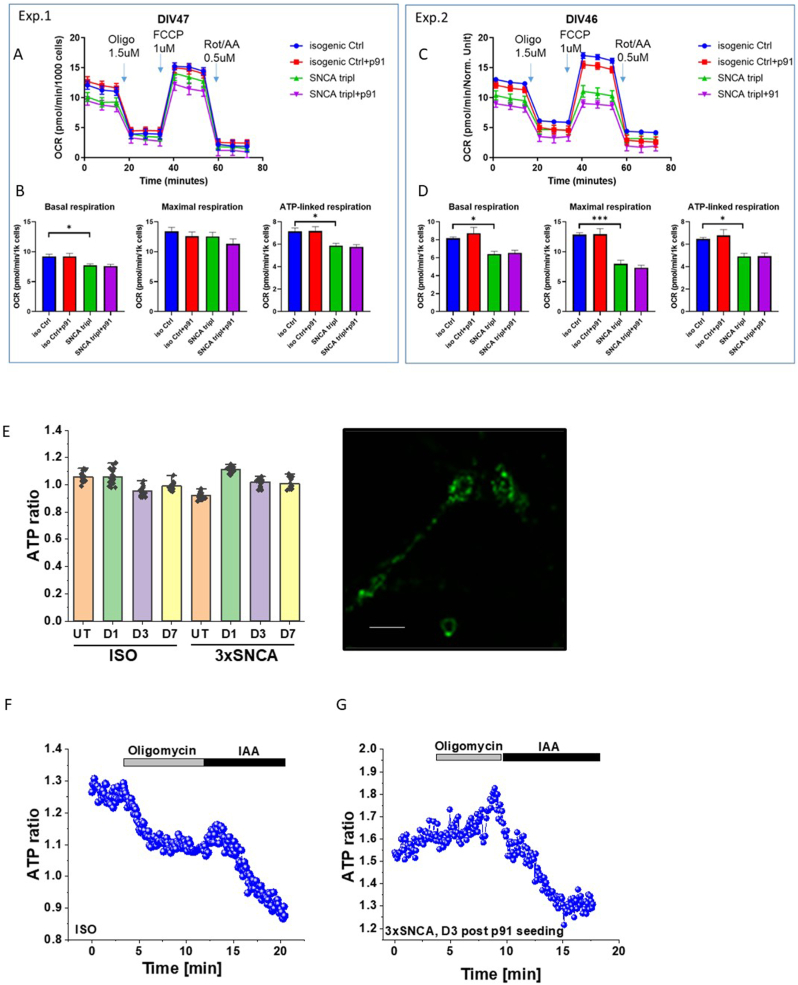
**3-week-p91 α-synuclein-seeding does not change cell respiration and ATP levels in control and 3xSNCA human iPSC-derived neurons**. Oxygen consumption rate (OCR) traces for isogenic control and SNCA triplication lines in presence/absence of p91 fibrils, measured using a Seahorse XF Analyzer (upper panel). Results from two independent experiments are presented. The injection order of Oligomycin (oligo), FCCP and Rotenone/Antimycin A (Rot/AA) is indicated (A, C). Mean ± SEM are shown for basal, maximal respiration and ATP-linked respiration for each of conditions (B, D). Number of replicates per condition n = 12 (experiment 1, left) and n = 15 (experiment 2, right). Effect of p91 fibrillar α-synuclein on the level of ATP in human neurons with SNCA triplication and isogenic control neurons (E). incubation of human iPSC-derived SNCA triplication and isogenic control (ISO) neurons with p91 fibrils had no effect on the basal ATP level (AT1.03 FRET ratio) but changed the mechanism of ATP production (F, G). Oligomycin (2 μg/mL), IAA 20 μM. Each measurement is represented by a dot. Values are mean ± SEM of N = 3 independent differentiation batches (n = 30 ROI for each group). Statistical analysis is performed using one-way ANOVA followed by Tukey post-test. Scale bars, 20 μm ∗p < 0.05, ∗∗p < 0.01, ∗∗∗p < 0.0001.

To investigate the effects of aSyn aggregation we measured oxygen consumption rate (OCR) in iPSC-derived neurons exposed to αSyn p91 fibrils using Seahorse XF Analyzer. SNCA triplication and control neurons were treated with p91 fibrils for 3 weeks, and OCR measurements were performed 25 days after fibrils exposure (Fig. 4A).

A significant decrease in both basal and ATP-linked respiration was observed in SNCA triplication line compared to isogenic control (Fig. 4A–D). Additionally, in one of the experiments a significant decrease in maximal respiration in SNCA triplication compared to isogenic control was also measured. At this time point, p91 fibrils did not induce changes in any of the parameters of mitochondrial respiration, neither in isogenic control nor in SNCA triplication line (Fig. 4C–D).

Although incubation of neurons with p91 fibrils had an effect on Δψm, it had no significant effect on the basal level of ATP neither in isogenic control neurons nor in neurons with SNCA triplication (Fig. 4E). Genetically-encoded ATP indicator (AT1.03) allows for the measurement of ATP in single cells. We estimated the effect of p91 incubation on two major ATP producers – oxidative phosphorylation (inhibited by oligomycin) and glycolysis (inhibited by iodoacetic acid (IAA)) using AT1.03. The application of oligomycin to isogenic control neurons expectably reduced the level of ATP in these neurons (Fig. 4F) and the subsequent addition of IAA induced further decrease in [ATP] in these cells. In contrast, inhibition of oxidative phosphorylation via oligomycin in SNCA neurons following 3 days p91 fibril incubation stimulated a rise in ATP levels (Fig. 3G) upon oligomycin application, in agreement with our Δψm results (Fig. 2). This further suggests the consumption of ATP by F0–F1-ATPase instead of its production. Application of glycolysis inhibitor IAA to these cells induced a profound decrease in [ATP] suggesting that ATP is mainly produced by the process of glycolysis rather than respiration (Fig. 4G).

### 3.5 p91 α-synuclein fibrils induced early mitochondrial fragmentation post application

**Fig. 5:**
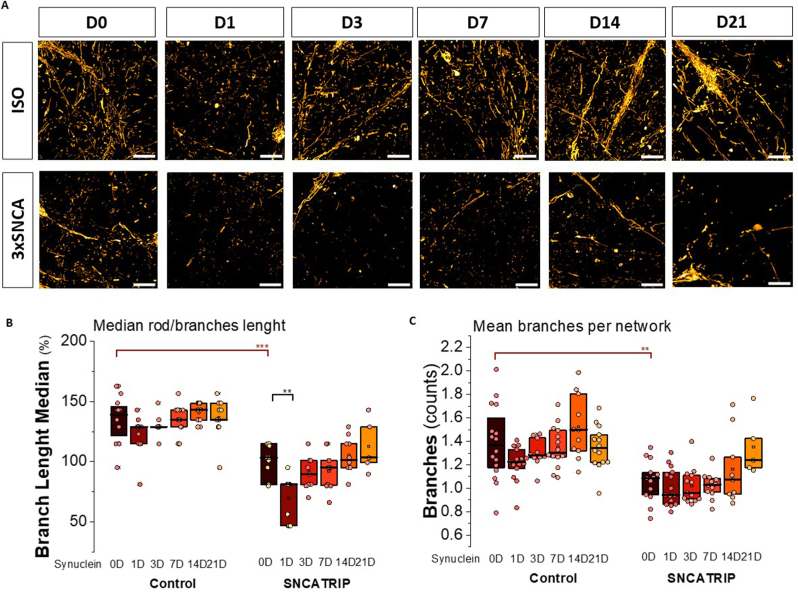
**Mitochondrial fragmentation under treatment with aSyn p91 fibrils.** A) TMRM images of cortical human neurons exposed to fibrillar aSyn p91 fibrils for 1, 3, 7, 14 or 21 days. Scale bar: 10 μm. B) Median length (% of 3xSNCA at day 0) of mitochondrial rods and branches in axons and C) number of branches per mitochondrial network were analysed in a total of 5–16 images per condition. Each dot shows the median or mean of all the axonal mitochondria in each image (neuronal bodies were excluded). Box plots represent the median and 25 and 75 percentiles. Non-parametric Kruskal-Wallis ANOVA with post-hoc Dunn's test for each group, Mann-Whitney for ISO vs 3xSNCA at D0, ∗∗p < 0.01, ∗∗∗p < 0.0001.

Analysis of the images of the TMRM fluorescence showed that treatment of the cells with p91 fibrils induced significant changes in the shape of the mitochondria with further fragmentation of the mitochondrial network after one day of treatment (Fig. 5A–C). This agrees with previous observations we made using hiPSCs-derived cortical neurons exposed to other aSyn fibrillar polymorphs [8]. However, longer incubation with the p91 fibrils led to the recovery of the mitochondrial shape (Fig. 5B and C). Thus, sophisticating mechanism of the maintenance of mitochondrial structure also can be affected by acute effect of fibrillar α-synuclein while prolonged incubation not enhance it and even lead to restoration of mitochondrial shape in neurons.

### 3.6 Short application but not seeding of p91 changes redox balance of human neurons

To study the effect of acute application of p91 fibrils on ROS production we assessed the level of hydrogen peroxide (H_2_O_2_) production using the Hyper-3 genetically-encoded quantitative probe.

**Fig. 6:**
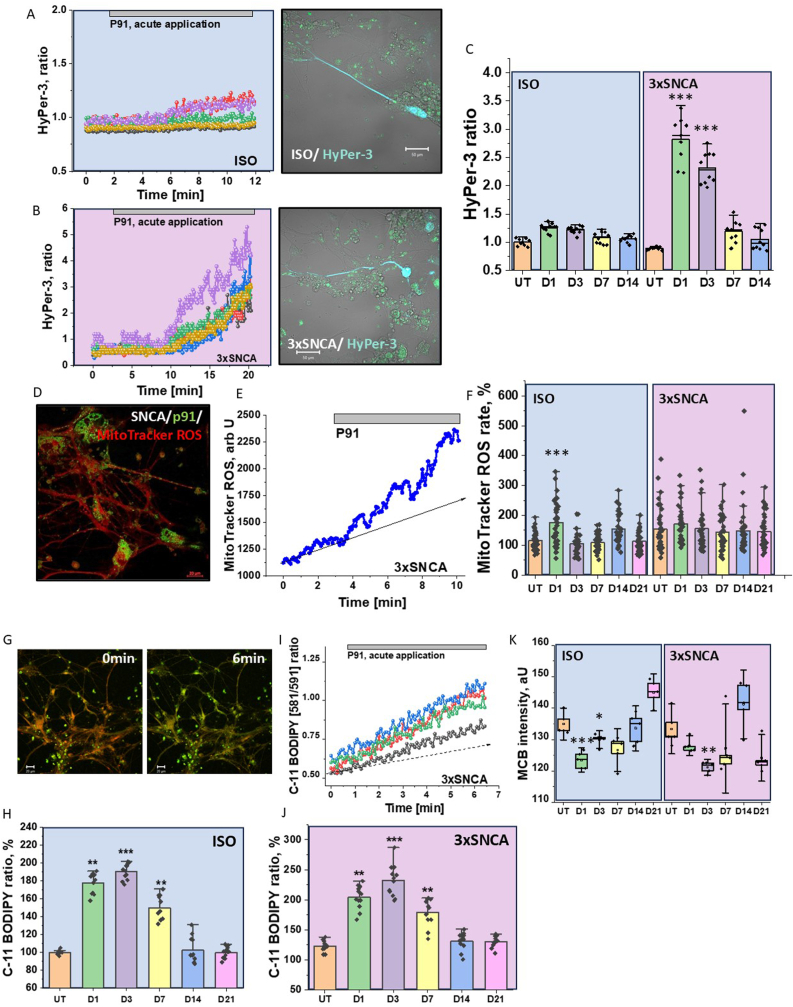
**Short application but not seeding of p91 changes redox balance of the human neurons.** ISO and 3xSNCA neurons were transfected with the HyPer-3 construct and H_2_O_2_ production rate upon acute application of p91 fibrils was measured and recorded (**A, B** representative traces and images). **(C)** Quantification bar chart of H_2_O_2_ production rate in ISO and 3xSNCA neurons over time after p91 seeding. Bars indicate the incubation period (D1,D3,D7,D14 and D21). N = 3 differentiation batches. (n = 30 ROI for each group). Error bars indicate SEM. Statistical analysis is performed using one-way ANOVA followed by Tukey post-test. Scale bars, 20 μm ∗p < 0.05, ∗∗p < 0.01, ∗∗∗p < 0.0001.**D,** Representative image of iPSC-derived ISO neurons labelled with MitoTracker red CM-H(2)X ROS. (**E)** Representative trace of mitochondrial ROS production rate in ISO control neuron upon acute p91 fibril exposure; **F**) Quantification of mitochondrial ROS production rates in control ISO and 3xSNCA neurons. Bars indicate the incubation period of p91 fibrils. Bars indicate the incubation period (D1,D3,D7,D14 and D21). N = 3 differentiation batches. (n = 30 ROI for each group). Error bars indicate SEM. Statistical analysis is performed using one-way ANOVA followed by Tukey post-test. Scale bars, 20 μm ∗p < 0.05, ∗∗p < 0.01, ∗∗∗p < 0.0001. **G,** Control ISO neurons were loaded with C11-Bodipy and lipid oxidation was recorded. **I,** Representative trace of p91-fibril-induced lipid peroxidation in control iPSC-derived neurons. **H,** Quantification of lipid peroxidation rates in ISO (**H**) and 3xSNCA(**J**) neurons preincubated with p91 fibrils 1, 3,7,14 and 21 days, respectively. N = 3 differentiation batches. (n = 50 ROI for each group). Error bars indicate SEM. Statistical analysis is performed using one-way ANOVA followed by Tukey post-test. Scale bars, 20 μm ∗p < 0.05, ∗∗p < 0.01, ∗∗∗p < 0.0001. **K** depicts GSH levels of ISO, compared to 3xSNCA neurons after seeding with p91 fibrils for 24 h; the levels of reduced GSH were assayed using MCB. Values are mean ± SEM of N = 3 differentiation batches (n = 50 fields for each group, automatic counts). Box plots and bars represent the median and 25 and 75 percentiles. Statistical analysis is performed using one-way ANOVA followed by Tukey post-test. Scale bars, 20 μm ∗p < 0.05, ∗∗p < 0.01, ∗∗∗p < 0.0001.

HyPer-3 probe is excited at 420 nm in its reduced state. When protons bind to the probe it becomes oxidised and can be excited at 500 nm. We tested the effect of acute addition of 0.75 μM p91 fibrils on the level of hydrogen peroxide in neurons with SNCA triplication and compared them to isogenic control neurons (Fig. 6A–B). We found that fibrillar α-synuclein induced only a small increase in the HyPer-3 ratio in isogenic control neurons, while in neurons with SNCA triplication, p91 fibrils induced a 3-fold increase in hydrogen peroxide production (Fig. 6A and B). Importantly, this effect was also observed in neurons with SNCA triplication during the first 72 h following p91 treatment and reverted to basal levels 7–14 days after treatment (Fig. 6C).

Changes in mitochondrial membrane potential can lead to elevation of ROS production in mitochondria [32]. We also used a mitochondrial ROS indicator MitoTracker red CM-H(2)X ROS which resulted in good localization of the fluorescent indicator in the mitochondria of human iPSC-derived neurons (Fig. 6 D, [11]. Acute application of p91 increased the rate of mitochondrial ROS generation (Fig. 6 E). However, 24 h incubation of ISO control with p91 induced 1.6-fold increase in the rate of MitoTracker red CM-H(2)X ROS fluorescence which was recovered to control values on the 3rd and the following days of incubation (Fig. 6 F). Although the initial basal level of mitochondrial ROS was higher in neurons with SNCA triplication, incubation of these cells with p91 fibrils induced only small changes after 24 h which did not change in further days of measurements (Fig. 6 F). Thus, acute application of p91 can induce short term increase in mitochondrial ROS production.

α-synuclein was shown to be able to induce lipid peroxidation and ferroptosis [9,10,33]. In agreement with this we found that application of p91 fibrils induce an acute increase in the rate of lipid peroxidation (Fig. 6G and H) that was also significantly higher in ISO control and 3xSNCA human neurons after 1, 3 and 7 days of incubation (Fig. 6I and J). However, the rate of lipid peroxidation in these cells recovered to basal after 14 days of p91 application (Fig. 6I and J) that confirms the importance of acute application of α-synuclein for generation of lipid peroxidation and no effect of p91 seeding on this process.

In order to understand whether p91-induced ROS production can change redox balance of neurons and induce oxidative stress we measured the level of the major endogenous antioxidant in brain – glutathione (GSH) using the fluorescent indicator monochlorobimane (MCB). We have found that incubation of the cells with p91 induced significant decrease of GSH level in both ISO control and SNCA triplication neurons after 24 h and 3 and 7 days of incubation but was recovering to control values after 14 and 21 days of incubation (Fig. 6 K). Thus, p91 fibrils could induce transient oxidative stress in human neurons the first days of application but this was not the case with seeding that mainly appeared after 3–7 days of incubation of cells with p91.

### 3.7 SNCA triplication, but not seeded fibrillar aSyn, induces neuronal cell death

**Fig. 7:**
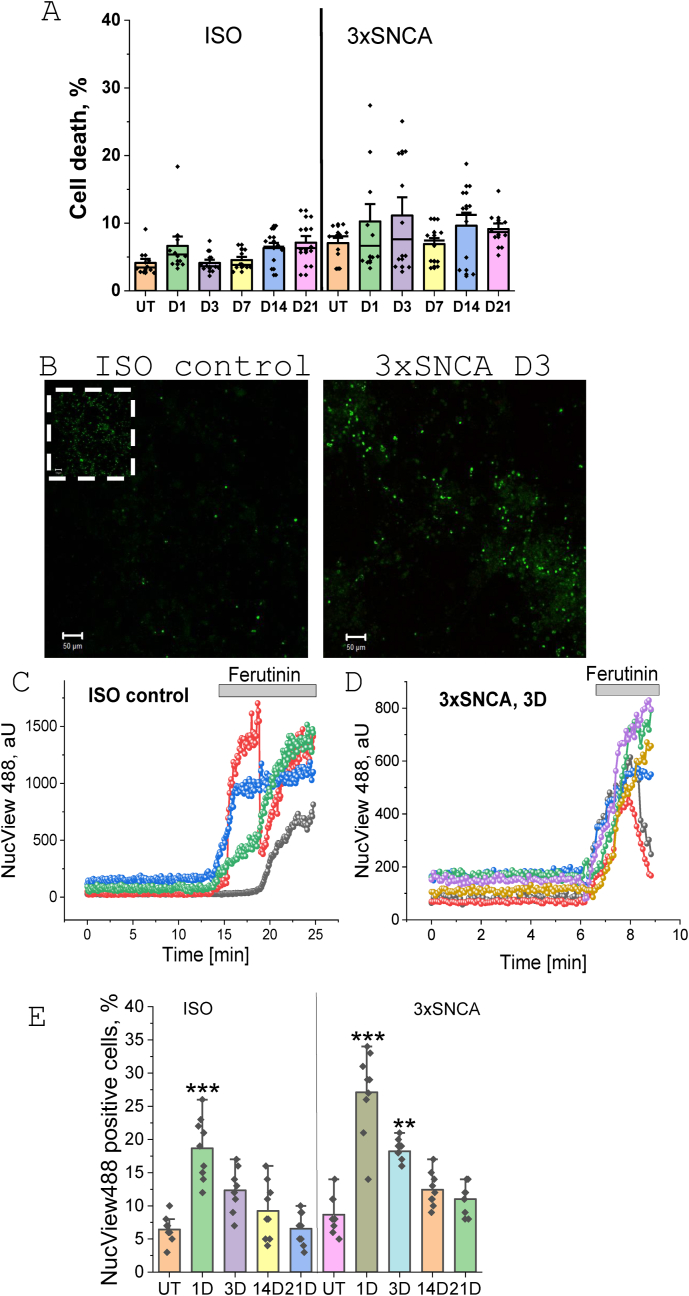
**SNCA triplication, but not seeded fibrillar aSyn, induces neuronal cell death.** Human IPSC-derived cortical neurons were loaded with Hoechst (live cells) and SYTOX green (dead cells). Neurons were exposed to p91 fibrillar αSyn for 24 h and cell survival measured in untreated and seeded neurons at days 1,3,7,14,21, respectively. (**A**) Quantification bar chart depicting (caspase-3 activation) in untreated and p91 fibril exposed ISO and 3xSNCA neurons. Bars indicate the mean values and error bars indicate SEM of n = 100 fields for each group (automatic counts) from N = 3 differentiation batches. **B**- representative images of the NucView488 fluorescence in ISO control or 3xSNCA neurons 3 days after p91 fibrils application. **C, D**- Application of 100 μM Ferutinin induces activation of caspase-3, cells with Nucview488 fluorescence were taken as 100 %. **E**-Percentage of NucView488-positive ISO control or 3xSNCA neurons at days 1,3,14,21 after exposure of cells to p91 fibrillar αSyn. Statistical analysis is performed using one-way ANOVA followed by Tukey post-test. Scale bars, 20 μm ∗p < 0.05, ∗∗p < 0.01, ∗∗∗p < 0.0001.

In order to study the effect of aSyn seeding on cell death we measured the percentage of dead neurons in isogenic control and SNCA triplication neurons before and 1, 3-, 7-, 14- and 21-days post-application of p91. We have found that in agreement with previously published data [9,15], SNCA triplication exhibits higher neuronal cell death compared to isogenic controls at basal and in all other conditions we have studied (Fig. 7 A). However, application of p91 and α-synuclein seeding had no significant effect on the percentage of necrotic cells in isogenic control or SNCA triplication neurons (Fig. 7A). Thus, seeding of neurons with fibrillar p91 α-synuclein does not induce necrotic cell death.

However, aggregated α-synuclein can induce apoptotic cell death by the activation of mitochondrial permeability transition pore opening [15,34]. We used the fluorescent substrate for caspase-3 – NucView488 to detect the effect of acute application of p91 fibrils or seeding of this α-synuclein on the induction of apoptotic cell death (Fig. 7B). In order to estimate the total number of cells and to calculate the percentage of neurons with caspase-3 activation, apoptosis was induced by application of calcium ionophore 100 μM Ferutinin [25,26](Fig. 7C–D). It should be noted that both iPSC derived neuronal lines had a basal level of 5–8 % cells with activated caspase-3 (Fig. 7E). Application of p91 induced significant increase in the number of apoptotic neurons in day 1 and 3 post-application in isogenic control and SNCA triplication neurons (up to 17 % in ISO; N = 9 experiments; p < 0.001; up to 28 % in 3xSNCA; N = 9 experiments; p < 0.001; Fig. 7 E) but the level of NucView488-positive cells has returned to control values in the days 14 and 21. This strongly suggests that application of exogenous p91 α-synuclein fibrils induces apoptosis in the first days after application followed by a recovery of the cells in the time of increased seeding of α-synuclein. Thus, seeded fibrillar α-synuclein does not induce apoptosis.

## 4 Discussion

Different species of alpha-synuclein are constantly generated through the aggregation from monomeric to fibrillar species. We know that all these aSyn species differ in size and shape, but also in structural content (alpha-helix vs. beta sheet rich), hydrophobicity, and solubility. Furthermore, they also show different functional properties based on their structure [35,36]. Some alpha-synuclein species are responsible specifically for seeding [37]. Seeding has been proposed to be the major mechanism by which aSyn pathological species spread through the brain and induce aggregation in connected neuronal populations, giving rise to the appearance of spread along a spatiotemporal gradient in a postmortem brain [38].

However, the role of seeding, and how it may drive the pathogenesis of Parkinson's disease, remains elusive. Seeds are detected in a range of biological fluids using the novel seed amplification assays in CSF that are being used for diagnosis in patients with Parkinson's disease, and seeding can be shown to occur *in vitro* and *in vivo* [39]. Here we addressed whether seeding occurs in human neurons, or whether seeds can drive synucleinopathy, and critically, whether seeds, and the seeding process, contribute to cellular toxicity.

The ability of various forms of aSyn to penetrate and distribute inside the cells is known already for a decade [40]. Here we found that labelled fibrillar aSyn is not only able to penetrate human neurons but can also be retained in these cells for the 21 days of observation, although the level of these fibrils gradually decreased over time. However, this distribution of the fibrillar aSyn in neurons over time allows us to document their effects in different days after their application. In agreement with previously published data [8,19,41,42], this penetration of fibrillar aSyn into the cells induces seeding which we could monitor using anti-phosphorylated aSyn antibodies (Fig. 1). The seeding propensity of the exogenous fibrils increased with time within the timeframe day 1–21.

On the basis of our experiments, on one hand we can conclude that most of the pathological effects of aSyn were observed in the first week after fibril application that suggests direct effects of the p91 fibrils on mitochondrial metabolism. On the other hand, considering that the level of seeding is maximal in neurons after 14 and 21 days of p91 application with minimal level of fluorescent p91 levels, the results from these days could be taken as a result of seeding or aSyn levels produced by this seeding. Thus, almost all our results show that seeding of p91 fibrils have a minimal to no effect on mitochondrial metabolism, redox balance and cell death. Importantly, seeding had no effect on both – isogenic control neurons and 3xSNCA neurons. Moreover, endogenous pathology of neurons with aSyn triplication was higher than the one induced by p91.

Thus, fibrillar seeding of aSyn has no implication into mitochondrial dysfunction and neuronal loss. We can suggest that if fibrils are being partially disintegrated withing several days to the oligomeric form of aSyn, this could potentially induce the acute pathology. However, oligomeric aSyn is quite toxic, so it in turn is possibly transforming itself to fibrillar form in the following days without the initiation of oligomer-specific seeding [37].

Previously we have shown that monomeric α-synuclein plays a physiological role on the F0–F1-ATPase activity and pathological species of aSyn also bind to this enzyme that leads to oxidation and triggers cell death [4,15]. In agreement to that, here we found that at day 3 fibrillar p91 α-synuclein induced not only changes in mitochondrial membrane potential, and NADH levels but also changed the activity of F0–F1-ATP synthase to ATPase mode to consume ATP for the maintenance of Δψm. However, it should be noted that this pathology is transient, and mitochondrial function recovers in control neurons with p91 seeding over the following weeks.

Fibrillar p91 was able to change mitochondrial membrane potential, mitochondrial NADH redox index that led to the acute elevation of ROS production in mitochondria and in the cytosol leading even to more increased rate of lipid peroxidation that is also in agreement with previously shown data [9,11,14]. However, it should be noted that these changes in redox balance did not induce cell death even under acute p91 application that confirms that fibrillar aSyn is mainly non-toxic to neurons and that seeding aggregation of aSyn does not induce pathology *per se*.

Surprisingly, the presence of fibrils long term is not associated with mitochondrial dysfunction even when they are not fully cleared. Furthermore, even once they have seeded the aggregation of endogenous aSyn, the neuron has the capacity to recover its mitochondrial function and remain viable.

The limitation of this study is coming from the advantage of using cultured human iPSC-derived neurons, which may be not fully functional compared to neurons *in vivo*. However, this stable human neuronal culture allowed us to produce these sophisticated, prolonged (more than 3 weeks) experiments which would not be possible in primary rodent neuronal cultures which are functional for much shorter times and functionality changing with the time (days *in vitro)* [43]. These prolonged experiments helped to separate an acute, transient toxic insult followed by a remarkable period of adaptation and functional recovery, highlighting the resilience of human neurons.

Thus, our observations identify an initial vulnerability to the presence of protein aggregates (fibrils), although this is followed by recovery despite ongoing seeding occurring. This leads us to the conclusion that all seeds, oligomeric or fibrillar, can interact with, and cause bioenergetic deficits and ROS generation in mitochondria. Certain species (oligomers) can also trigger cell death through mPTP opening or through ferroptosis. Fibrils, on the other hand, do not induce toxicity over three weeks of seeding. The process of seeding aggregation itself is therefore not toxic to the neuron in this experimental paradigm and therefore is unlikely to be the main driver of pathogenesis, and in particular the cause of neuronal cell death in the cortex.

## CRediT authorship contribution statement

**Plamena R. Angelova:** Writing – review & editing, Writing – original draft, Methodology, Investigation, Formal analysis, Data curation, Conceptualization. **Noemi Esteras:** Writing – review & editing, Writing – original draft, Investigation, Formal analysis, Data curation. **James Evans:** Writing – review & editing, Writing – original draft, Investigation, Formal analysis. **Marko Kostic:** Writing – review & editing, Writing – original draft, Investigation, Formal analysis. **Ronald Melki:** Writing – review & editing, Resources, Methodology, Funding acquisition, Conceptualization. **Jochen H.M. Prehn:** Writing – review & editing, Supervision, Resources, Project administration, Conceptualization. **Sonia Gandhi:** Project administration, Funding acquisition, Conceptualization. **Andrey Y. Abramov:** Writing – review & editing, Writing – original draft, Supervision, Methodology, Investigation, Conceptualization.

## Funding

This study has been funded by Mitochondrial Dysfunction in Parkinson's Consortium (PD-MitoQUANT). PD-MitoQUANT has received funding from the 10.13039/501100010767Innovative Medicines Initiative 2 Joint Undertaking under grant agreement No. 821522. This Joint Undertaking receives support from the European Union's 10.13039/501100007601Horizon 2020 research and innovation program and 10.13039/100013322EFPIA. NEG is supported by a Ramon y Cajal Fellowship (RYC2021-034267-I) funded by 10.13039/501100004837Spanish Ministry of Science and Innovation 10.13039/501100004837MCIN/10.13039/501100011033AEI/10.13039/501100011033 and the 10.13039/501100000780European Union “NextGenerationEU”/PRTR, and a grant from PID2022-137011OA-I00/10.13039/501100011033AEI/10.13039/501100011033/10.13039/501100002924FEDER, EU.

## Declaration of competing interest

The authors declare that they have no known competing financial interests or personal relationships that could have appeared to influence the work reported in this paper.

The author is an Editorial Board Member/Editor-in-Chief/Associate Editor/Guest Editor and was not involved in the editorial review or the decision to publish this article.

## Data Availability

Data will be made available on request.
